# The midwife’s role in achieving the Sustainable Development Goals: protect and invest together – the Swedish example

**DOI:** 10.1080/16549716.2022.2051222

**Published:** 2022-05-06

**Authors:** Helena Lindgren, Malin Bogren, Ingrid Osika Friberg, Marie Berg, Gabriella Hök, Kerstin Erlandsson

**Affiliations:** a Department of Women’s and Children’s Health, Karolinska Institutet, Solna, Sweden; b Institute for Health and Care Science, Sahlgrenska Academy, Gothenburg, Sweden; c Swedish Agency for Gender Equality, Gothenburg, Sweden; d Swedish Institute for Global Health Transformation, Stockholm, Sweden; e Department of Health, Care and Wellbeing, Dalarna University, Falun, Sweden

**Keywords:** Midwifery, SDG, agenda 2030, Sweden, global

## Abstract

‘The midwife’s role in achieving the Sustainable Development Goals: Protect and Invest Together’ is a report providing the reader the opportunity for understanding and appreciating the history of midwifery in Sweden and the interlinked nature of the United Nation’s SDGs supporting health and wellbeing of women and children. To realise the opportunity to have a country with well-educated midwives of high academic standard, and, at the same time, promoting gender equality and equity we need to protect and invest together in midwives. This paper provides the foundation for a revitalised discussion on midwives’ role for women and child health in the 21st century. The full Swedish Midwifery report was published in October 2021.

## Background

The UN Millennium Declaration and the Millennium Development Goals (MDGs) were adopted in 2000, and global efforts were made to achieve the eight goals agreed upon for the first 15 years of the new millennium. These efforts became the foundation for the development of the Sustainable Development Goals (SDGs) adopted by the leaders of the world in 2015. Sweden responded to the international agenda for global development by translating it into national policy. One priority in working toward achieving the MDGs was the empowerment of women. Sexual and reproductive health and rights, the promotion of gender equality, and addressing the imbalance of power are subjects that must be taken into account in implementing the MDGs. Advancing the United Nation’s 2030 Agenda in Sweden might call for letting the past shed light on the futureby looking at the history of midwifery in Sweden.

Already in 1886, Swedish midwives had organized themselves into a joint union, the Swedish Midwifery Association [1]. Issues such as salary, pension, and quality of midwifery education were placed on the agenda. The midwives’ drive and desire to advance societal development led to the establishment of free maternity care in Sweden during the 1930s. Contraceptive counseling and giving the midwive’s responsibility forduring the same period were important. After this efforts began to provide quality abortion care. The development of midwifery was accompanied by a decrease in maternal and newborn mortality in Sweden [2]. Three current reports show the potential for welll organized midwifery programs to to ensure the health and wellbeing of the next generation women and girls [3–5]. The health and wellbeing of women and children form the core of what will become society’s future human capital [6]. Sweden was one of the first countries in the world to make midwifery into a medical profession that required education sufficient to ensure that midwives were qualified to give care previously not available for women in general.

Through a collaboration between the Swedish Institute for Global Health Transformation (SIGHT) and the Swedish Association of Midwives, a review of the role of midwifery in the implementation of the UN 2030 Agenda in Sweden resulted in The Swedish Midwifery Report 2021.

**Figure 1.**
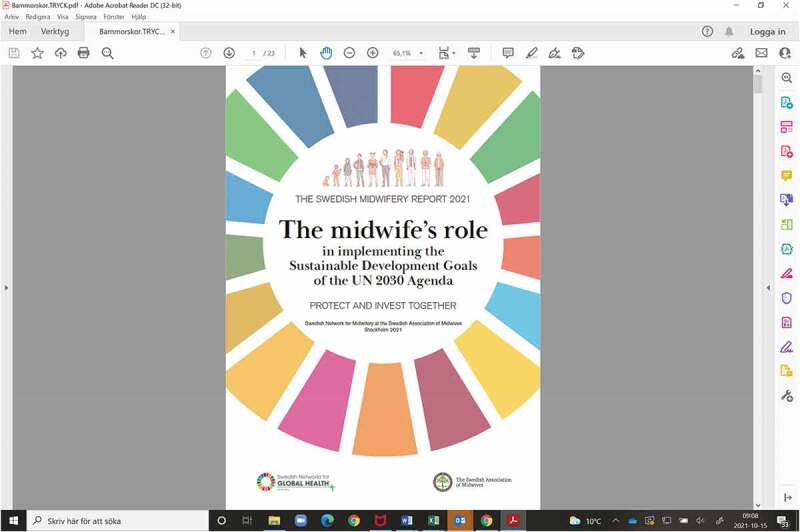
The report The midwife's role in implementing the Sustainable Development Goals of the UN 2030 Agenda was launched in October 2021.

Figure 1 here. The full report is available at the website https://sight.nu/swedish-network-for-midwifery/

The initial initiative leading to this report comprised round table discussions on the Role of the midwife in Global Health. These were organised by the Swedish Association of Midwives, through its International Council and were hosted at the Swedish Ministry for Foreign Affairs (MFA) in 2018 and 2019. This led to the formation of the Swedish Midwifery Network in 2020. The purpose of this network is to bring together actors who together can increase the impact of Swedish models on on Global Health.

The Swedish midwife works at all levels in a hub-and-spoke health-care system that consists of network with a tertiary level hospital in the center, referred to as a hub, a hospital thatoffers a full array of services. This hospital is complemented by secondary health facilities referred to as spokes. These offer more limited-service referring women and children in need of more specialised services to the hub for treatment and care [4]. Continuous meetings and discussion within the writing group that prepared this report led to an analysis on how the 17 SDGs named in the UN 2030 Agenda for Sustainable Development could guide the use of midwifery as a driving force for policy change. Midwives were a low-cost workforce who could do their work all over the country.

## The report

The Swedish Midwifery report describes the history of midwifery in Sweden, a history that can be presented as consisting of three main parts

1. *The development of the role of the midwife in Sweden –* From traditional birth attendants to licensed midwives with an academic degree, a process that led to progressprofessionalization and academisation of the Swedish midwife.
2. *Sweden’s response to the SDGs relevant to Swedish midwifery practice* – a) **SDG 2 Zero Hunger**. Without adequate and sustained investments in good nutrition for girls, women, and children, the SDGs will not be realised. Malnutrition will represent an often invisible impediment to the achievement of good health (SDG 3). Malnutrition not only from a lack of sufficient nutritious food, but also from a host of intertwined factors linking empowerment of women and families, health during pregnancy and childbirth, care for the newborn, breastfeeding, complementary food counseling to parents, clean water supplies, sanitation and hygiene with access to food and resources. b) **SDG 3** The goal of SDG 3 is to ensure healthy lives and promote well-being for all ages. The associated targets include reducing the global maternal mortality ratio and ending preventable deaths of newborns and children. Having access to a midwife is beyond doubt the single most important factor in reducing mortality and morbidity among women and children worldwide [3,5]. Midwives working in interdisciplinary teams, planning and providing care, and reaching out to consultants in interdisciplinary teams when needed can provide the most cost-effective care but also the core of a model that shows the best outcomes and highest levels of satisfaction among women. c) **SDG 4** Quality Education, the first education programme for midwives in Sweden started in the 1700s. Today all midwifery education programmes in Sweden, and the full masters programme are based on a ‘midwifery discipline’ labeled ‘sexual, reproductive and perinatal health’ or similar. These can serve as a model d) **SDG 5** Gender equality. Gender equality empowers all women and girls. Gender equality is a necessary foundation for a peaceful, prosperous, and sustainable world and leads to economic growth and development. In October 2014, Sweden became the first country in the world to launch a feminist foreign policy. This means applying a systematic gender equality perspective throughout the whole foreign policy agenda. The policy work is organised around rights, representation, and resources. It is based on the premise that gender equality is not just a women’s issue – it benefits everyone. As a means of the feminist policy Sweden has campaigned for women’s and girls’ sexual and reproductive health and rights, and greater access to midwives worldwide. In Sweden, the total per capita cost for health care is 20% higher for women than for men (and the difference is largest for primary care and smallest for inpatient care). When excluding health care for reproduction and sex-specific morbidity from total health care cost, the cost difference between women and men declines to 8%. A separate budget track for reproductive health would facilitate the investment in midwives, being the profession that meets most of the reproductive needs. The total cost for care received in connection with reproduction and sex-specific morbidity is estimated to be 7.7% of the total health care budget [7]. e) **SDG 16** Promote peaceful and inclusive societies for sustainable development, provide access to justice for all, and build effective, accountable, and inclusive institutions at all levels. The tax-funded, free-of-charge, and evidence-based health care system in Sweden is a prerequisite for the high attendance to maternal and child health care and the public trust in the system. According to Swedish health care reports, almost 100% of pregnant women utilise their right to ANC services. The routine of midwives reporting the birth of every child helps the delivery of birth certificates and has been in use for more than 200 years, placing Sweden as the country with one of the oldest and best functioning birth registration systems in the world. The Swedish model should be considered for use wherever possible. A birth certificate should be issued for every person born within a single country, and some form of data on every individual, no matter where they are born, should be kept in appropriate registers because such data finds extensive use in medical research. The feminist foreign policy and the collaboration between strong institutions constitutes a solid ground for midwives as well as other health care providers. f) **SDG 17** Partnerships for the goals describe the cooperation and partnerships with national and international civil society, multilateral organisations, public agencies, and the private sector. Sweden works for sustainable development and helps create conditions for people living in poverty and oppression to improve their living conditions nationally and internationally. For over 30 years, Sweden has been supporting the education of and accessibility to midwives in low- and middle-income countries. The unique tradition and experience of more than 300 years of midwifery in Sweden underpin Sweden’s capacity-building support.

## Conclusion (Part 3)

Efforts should be made to identify the nature of interlinkages between the SDGs and various elements of midwifery. In addition efforts should be made to PROTECT midwives’ rights, to continue academisation of the profession, provide decent work, and practice environments. INVEST in midwifery leadership to accelerate the implementation of the 2030 Agenda. TOGETHER, we all have a role to play to ensure that midwives are supported, protected, motivated, and equipped to always deliver safe health care.

## Paper context

- Provides an opportunity for understanding the linkage between the history of midwifery in Sweden and the selected SDG:s.
- The tax-funded health care system and the Swedish gender policy provides a foundation for equal access to quality midwifery care.
- There is a need to protect and invest in midwives in order to achieve the UN 2030 Sustainable Development Goals in Sweden and worldwide.
